# On the Coronavirus (COVID-19) Outbreak and the Smart City Network: Universal Data Sharing Standards Coupled with Artificial Intelligence (AI) to Benefit Urban Health Monitoring and Management

**DOI:** 10.3390/healthcare8010046

**Published:** 2020-02-27

**Authors:** Zaheer Allam, David S. Jones

**Affiliations:** 1 The Port Louis Development Initiative (PLDI), Port Louis 11302, Mauritius; 2 School of Architecture & Built Environment, Deakin University, Geelong, VIC 3220, Australia; david.jones@deakin.edu.au

**Keywords:** urban health, smart cities, artificial intelligence, Coronavirus, pandemics, future cities, Internet of Things (IoT), COVID-19, 2019-nCoV

## Abstract

As the Coronavirus (COVID-19) expands its impact from China, expanding its catchment into surrounding regions and other countries, increased national and international measures are being taken to contain the outbreak. The placing of entire cities in ‘lockdown’ directly affects urban economies on a multi-lateral level, including from social and economic standpoints. This is being emphasised as the outbreak gains ground in other countries, leading towards a global health emergency, and as global collaboration is sought in numerous quarters. However, while effective protocols in regard to the sharing of health data is emphasised, urban data, on the other hand, specifically relating to urban health and safe city concepts, is still viewed from a nationalist perspective as solely benefiting a nation’s economy and its economic and political influence. This perspective paper, written one month after detection and during the outbreak, surveys the virus outbreak from an urban standpoint and advances how smart city networks should work towards enhancing standardization protocols for increased data sharing in the event of outbreaks or disasters, leading to better global understanding and management of the same.

## 1. Introduction

The novel Coronavirus outbreak, (previously known as the 2019-nCoV and later renamed COVID-19 during the writing of this manuscript) is leading to the closure of entire cities in China, and causing stringent measures to be taken in others. While in distant different continents, far from China where the virus was first reported, places are being placed on high alert. In Wuhan, where the virus broke, schools, roads and markets have been shut down [1]. The same is true in Hong Kong, Beijing and Hubei Province amongst surrounding areas, as precautionary measures are being emphasized to ensure that the spread of the virus is minimized, and complete and accurate information on the virus is being obtained [2]. However, the rate of spread of the virus and the uncertainties surrounding the entire situation has led the World Health Organization (WHO) on 30 January 2019 to declare the Coronavirus outbreak a ‘Global Public Health Emergency’. WHO determined, however, not to declare the outbreak a ‘Public Health Emergency of International Concern’ (PHEIC) which is a higher level of declaration. A PHEIC is defined as “an extraordinary event which is determined to constitute a public health risk to other States through the international spread of disease and to potentially require a coordinated international response” whose scope may include: serious, sudden, unusual or unexpected; carries implications for public health beyond the affected State’s national border; and may require immediate international action [3].

With the world having experienced some notable influenza pandemics in the past, a Global Initiative on Sharing All Influenza Data (GISAID) platform [4] was established and was instrumental in the rapid sharing of information by the Chinese scientists regarding the emergence of the COVID-19 virus. Through this platform, scientists from other regions were observed to gain access to information and are, subsequently, able to act in a much faster capacity; like in the case of scientists from the Virus Identification Laboratory based at Doherty Institute, Australia, who managed to grow a similar virus in the laboratory after accessing the data shared by the Chinese scientists [5].

Beyond the aspect of pandemic preparedness and response, the case of COVID-19 virus and its spread provide a fascinating case study for the thematics of urban health. Here, as technological tools and laboratories around the world share data and collectively work to devise tools and cures, similar efforts should be considered between smart city professionals on how collaborative strategies could allow for the maximization of public safety on such and similar scenarios. This is valid as smart cities host a rich array of technological products [6,7] that can assist in early detection of outbreaks; either through thermal cameras or Internet of Things (IoT) sensors, and early discussions could render efforts towards better management of similar situations in case of future potential outbreaks, and to improve the health fabric of cities generally. While thermal cameras are not sufficient on their own for the detection of pandemics -like the case of the COVID-19, the integration of such products with artificial intelligence (AI) can provide added benefits. The fact that initial screenings of temperature is being pursued for the case of the COVID-19 at airports and in areas of mass convergence is a testament to its potential in an automated fashion. Kamel Boulos et al. [8] supports that data from various technological products can help enrich health databases, provide more accurate, efficient, comprehensive and real-time information on outbreaks and their dispersal, thus aiding in the provision of better urban fabric risk management decisions.

The above improvements in the healthcare sector can only be achieved if different smart city products are fashioned to support standardized protocols that would allow for seamless communication between themselves. Weber and Podnar Žarko [9] suggest that IoT devices in use should support open protocols, and at the same time, the device provider should ensure that those fashioned uphold data integrity and safety during communication and transmission. Unfortunately, this has not been the case and, as Vermesan and Friess [10] explain, most smart city products use proprietary solutions that are only understood by the service providers. This situation often creates unnecessary fragmentation of information rendering only a partial integrated view on the dynamics of the urban realm. With restricted knowledge on emergent trends, urban managers cannot effectively take decisions to contain outbreaks and adequately act without compromising the social and economic integrity of their city. This paper, inspired by the case of the COVID-19 virus, explores how urban resilience can be further achieved, and outlines the importance of seeking standardization of communication across and between smart cities.

## 2. On the Prospects of Urban Health Data

With the advent of the digital age and the plethora of Internet of Things (IoT) devices it brings, there has been a substantial rise in the amount of data gathered by these devices in different sectors like transport, environment, entertainment, sport and health sectors, amongst others [11]. To put this into perspective, it is believed that by the end of 2020, over 2314 exabytes (1 exabyte = 1 billion gigabytes) of data will be generated globally [12] from the health sector. Stanford Medicine [12] acknowledges that this increase, especially in the medical field, is witnessing a proportional increase due to the increase in sources of data that are not limited to hospital records. Rather, the increase is being underpinned by drawing upon a myriad and increasing number of IoT smart devices, that are projected to exponentially increase the global healthcare market to a value of more than USD $543.3 billion by 2025 [13]. However, while the potential for the data market is understood, such issues like privacy of information, data protection and sharing, and obligatory requirements of healthcare management and monitoring, among others, are critical. Moreover, in the present case of the Coronavirus outbreak, this ought to be handled with care to avoid jeopardizing efforts already in place to combat the pandemic. On the foremost, since these cut across different countries, which are part of the global community and have their unique laws and regulations concerning issues mentioned above, it is paramount to observe them as per the dictate of their source country’s laws and regulations; hence, underlining the importance of working towards not only the promoting of data through its usage but also the need for standardized and universally agreed protocols.

While the significance of such data in advancing efficiency, productivity and processes in different sectors is being lauded, there are criticisms arising as to the nature of data collection, storage, management and accessibility by only a small group of users. The latter particularly includes select ICT corporations that are also located in specific geographies [6,14,15,16,17]. These criticisms are justified, as in recent years, big data is seen as the new ‘gold rush’ of the 21st century and limiting its access means higher economic returns and increased influence and control at various scales to those who control data. These associated benefits with big data are clearly influencing geopolitical standings, in both corporate and conventional governance realms, and there is increased competition between powerful economies to ensure that they have the maximum control of big data. As case in point is the amount of ‘push and pull’ that has arisen from Huawei’s 5G internet planned rollout [18]. Though the latter service offers unprecedented opportunities to increase internet speeds, and thereby influence the handling of big data, countries like the U.S. and some European countries that are key proponents and players in global political, economic and health landscapes, are against this rollout, arguing that it is a deceptive way of gathering private data under the guise of espionage. On this, it has been noted that the issue of data control and handling by a few corporations accords with their principles of nationalism, and that these work for their own wellbeing as well as to benefit the territories they are registered in. Therefore, geopolitical issues are expected on the technological front as most large data-rich corporations are located in powerful countries that have influence both economically, health-wise and politically [15,19,20]. Such are deemed prized tokens on the international landscape, and it is expected that these economies will continue to work towards their predominant control as much as possible. On the health sector, the same approach is being upheld where critical information and data are not freely shared between economies as that would be seen to be benefiting other in-competition economies, whereas different economies would cherish the maximization of benefits from such data collections. 

## 3. A High-Level Survey of the Coronavirus (COVID-19) Outbreak

In addition to the obvious deep-rooted social issues related to nationalism, other challenges include the increasing movement of people globally that is being enhanced by reduced costs and higher speed. In particular, these challenges are more pronounced when it comes to public health. This is because most of the health-related data collected not only can compromise local nations, but also captures those of travelers. In such cases, in a bid to improve the health status of a nation, it becomes paramount to factor in data from other regions necessitating unhindered sharing of this data. 

Such data-sharing truth is emphasized in situations like the recent case of Coronavirus outbreak threatening the global health environment, facilitated by air transportation. The virus was first reported in Wuhan, China, and in a matter of three weeks (by 17th January 2020) over 300 cases were confirmed in that region, and 10 days later (26th January 2020), a total of 2014 cases of Coronavirus have been reported, with 684 of those being confirmed, and with 29 reported outside China. The fatalities from the virus stands at 56 as of 26th January 2020 [21]. The virus had then been confirmed in various countries including Taiwan, South Korea, Japan, Thailand, France, the United States, Singapore and Vietnam [22]. 

In the above case, though major cities are known to prepare themselves for potential outbreaks, their health policies and protocols are observed to diverge from one another. Thus, without a global collaborative approach, progress towards working for a cure and universally acceptable policy approach can take longer. Such fears, of a lack of international collaboration, were highlighted by the World Health Organization (WHO) during an emergency meeting in Geneva on 22nd January 2020 to determine whether the virus outbreak had reached a level warranting international emergency concern. However, WHO was satisfied that China was being proactive in this case, unlike in 2002, when China withheld information on the outbreak for far too long, causing delays in addressing the epidemic [3]. As in this instance, it is the opinion in this paper that if there was seamless collaboration and seamless sharing of data between different cities, it would not warrant such a high-level meeting to result in action, and instead, a decision could have been made much earlier. On this, the saddest part is that some global cities are less prepared to handle the challenges posed by this type of outbreak for lack of information on issues like symptoms of the virus, the protective measures to be taken, and the treatment procedures that an infected person should be processed through, amongst other issues. 

The timely response by stakeholders in regard to this new outbreak are commendable compared to previous cases. The latter includes the Severe Acute Respiratory Syndrome (SARS) outbreak in 2002 that took substantial time (from November 2002 to April 2003) to identify and be dealt with [23]; the Ebola outbreak in West Africa in 2013 that took months to determine; and the Zika Virus that was first reported in 2014 before being successfully identified in 2015. 

With the Coronavirus (COVID-19), it took only 17 days (31st December 2019 to 17th January 2020) to be identified. The sharing of data has also been quicker, as immediately after the virus’ genetic sequence was discovered, Chinese scientists were able to share the information with the WHO, thus helping in its identification and enabling the auctioning of precautionary measures in other countries. Latest technological tools have also allowed for the receipt of information in real- time, in contrast to traditional epidemiological approaches that would have required months to identify the outbreak type [24]. Similarly, though substantial data and information on the disease has been shared, Wetsman [25] acknowledges that there is a lack of some vital information, like the ease of spread of the virus from person-to-person, and this is a key to containing the disease as interactions between people from different parts of the globe are still active. This hindrance can be made further possible as many cities advance in their smart and safe city model implementation towards constructing sufficient soft and hard urban infrastructures equipped with, for example, thermal imagery sensors to allow for early detections. However, while that is the case, data access to many is a challenge because the information is often seen as being sensitive for national security reasons, whilst at the same time, acknowledging that a virus outbreak is an equal threat to both national security and the economy.

## 4. The Urban Economy and Urban Safety

The outbreak of any disease has significant impacts on local economies across the globe. For instance, when SARS (Severe Acute Respiratory Syndrome) (SARS-CoV) broke in China in 2002, it was estimated, that the Asian region incurred tremendous negative impacts socially, health-wise and economically, potentially amounting to Asian regional economy losses of between USD $12–18 billion from tourism, travel and retail sales industries alone [26]. The Zika virus outbreak, spread by daytime-active Aedes mosquitoes, is estimated to have cost equator-belt local economies in affected areas between USD $7 and USD $18 billion [27]. The Ebola virus (or Ebola hemorrhagic fever (EHF)) caused an estimated loss of USD $2.2 billion in GDP in three West African economies (Guinea, Liberia and Sierra Leone) in 2015 alone [28]. In regard to the current epidemic of Coronavirus, though it is too early to quantify or project its impacts on the global economy, there are fears that it may take the precedent of other outbreaks where billions of dollars will be lost. The foundations for this escalating loss can be witnessed in the rapid growth of travel bans being enacted by some countries and their international airports, especially specifically restricting people from visiting the affected regions in China and their growth into general non-Chinese travel movements. On this, noting that the outbreak came almost on the eve of the Lunar New Year celebrations, and that it had been estimated that over 400 million people were expected to travel in different parts of the world and China to observe this festivity, the majority have had to reconsider their options as to flights, hotels and entertainment events due to service provider cancellations [29]. Those who had already booked their flights are expected to receive their refunds following the directive by the Civil Aviation Administration of China, however, this move has already affected the share value of Chinese airline companies [29]. 

The above impacts demonstrate that the issues of virus outbreaks transcend urban safety and impacts upon all other facets of our urban fabric. Therefore, it becomes paramount to ensure that the measures taken to contain a virus transcend nationalist agendas where data and information sharing is normally restricted, to a more global agenda where humanity and global order are encouraged. With such an approach, it would be easier to share urban health data across geographies to better monitor emerging health threats in order to provide more economic stability, thereby ensuring no disruptions on such sectors like tourism and travel industries, amongst others. This is possible by ensuring collaborative, proactive measures to control outbreak spread and thus, human movements. This would remove fears on travelers, and would have positive impacts upon the tourism industry, that has been seen to bear the economic brunt whenever such outbreaks occur. This can be achieved by ensuring that protocols on data sharing are calibrated to remove all hurdles pertaining to sharing of information. On this, Lawpoolsri et al. [30] posits that such issues, like transparency, timelessness of sharing and access and quality of data, should be upheld so that continuous monitoring and assessment can be pursued. 

## 5. Standardization and Data Sharing through the Smart City Network

Virus outbreaks in recent years have shown that, in the urban realm, data, including health data, can be sourced from diverse places. Presently, in the case of Coronavirus (COVID-19) outbreak, data is being collected from airports through screening and monitoring, through the use of smart sensors installed in airport infrastructures and from personnel working in those air/seaports. For instance, it has been reported that in the U.S.A., screening is being carried out at 20 different airports to ensure that possible affected people are intercepted for quarantine at the point of entry. Beside airports, as reported by Buckley and May [2], data is also being collected at bus terminals, market places (in Wuhan), subways, and also in health facilities where patients are taken for further medical attention. Such is prevalent especially in China, and other Asian regions where cases of the virus have been recorded and confirmed.

In addition to these methods, other smart city data sources include the application of terminal tracking systems that are mostly emphasized in Safe City concepts, where, at the point of entry or departure, relevant data is collected and analyzed. Li et al. [31] highlights that sensors installed in such locations have the potential to receive and distribute data in real-time to digital infrastructures within the network, and their interconnectedness in the network renders them extremely efficient in providing real-time updates on different issues. Urban areas are also known to be amassed with numerous Urban Health sensors, some of which are wearable. Though these are not specifically fashioned to track the present case of virus outbreak, they are able to track other related parameters like heartbeat, blood pressure, body temperature and others variables, that when analyzed can offer valuable insights. Loncar-Turukalo et al. [32] hail these devices for their role in transforming the health care sector especially by allowing for Connected Health (CH) care, where data collected from them can be analyzed and provide insightful information on the health scenario in any given area. Vashist et al. [33] further highlight how emerging features such as spatiotemporal mapping, remote monitoring and management, and enhanced cloud computing capabilities can emanate from such endeavours, leading to better urban management potential.

While it is true that the basic source of medical data is generally sourced from general practitioners or medical laboratories—a fact that has also been affirmed in the case of the current epidemic—this paper explores how data sourced from an urban perspective can contribute to the medical narrative. The conviction to dwell on the urban realm in this manuscript is based on the fact that the current epidemic (COVID-19) is transmitted majorly through human-to-human contact, and in most cases, especially where the spread is reported in a different country, the first point of contact is an urban area, where large groups of people convene, like airports or subway stations. In most cases, such facilities, which are mostly based in urban areas, are observed to have installed surveillance technologies to ensure that anyone showing any symptoms of the disease are identified and quarantined. However, even in such cases, as underlined in the present manuscript, the need for anonymizing medical data is emphasized to ensure that the use of current technologies does not breach data privacy and security requirements, across different geographies. In this case, novel technologies like Blockchain technologies and quantum cryptography can aid in the discussion and be made to integrate with data collecting technologies. This would render an increased wealth of data from both the medical field and smart city operators, while ensuring privacy and security; hence, aiding in providing relevant information for better informed decisions.

However, despite the indisputable roles that installed devices play in providing relevant health information, their data communication aspect needs to be reviewed. First, communications are seen to be geography-restricted (restricted to a given location), such that they seldom expand or communicate with their like, installed beyond their restricted areas. Secondly, these devices are usually sourced and installed by separate corporations that maintain unique and specific standards for data processing and sharing, and accordingly, tying cities to the sole usage of their product(s). Such strategies are adopted as private corporations try to maximize their economic gains, since the digital solution market is a lucrative one and is expected to continue growing and expanding [6,7].

For its current application, the standardization of protocols as elaborated in this manuscript need to be pursued to ensure that there is seamless sharing of information and data. By doing this, it is expected that issues like burdens of collecting data, accuracy and other complexity that are experienced (when systems are fragmented) are reduced or eliminated altogether. The standardization can be achieved by, for example, ensuring that all the devices and systems are linked into a single network, like was done in the U.S., where all the surveillance of healthcare were combined into the National Healthcare Safety Network (NHSH) [34]. The fact that cities are increasingly tuning on the concept of Smart Cities and boasting an increased adoption rate of technological and connected products, existing surveillance networks can be re-calibrated to make use of those new sets of databases. Appropriate protocols however have to be drafted to ensure effective actions while ensuring privacy and security of data and people.

With scenarios like the present Coronavirus (COVID-19) outbreak, that not only impacts upon the economic status of cities, but also affects their social standing, it becomes imperative to emphasize the adoption of universal standards for data sharing. Such a move could have far reaching impact across cities and territories especially in positively combating outbreaks and disasters in a quicker, safer and standardized way, such that when the cure is discovered, the results can be replicated in various parts of the globe. With a collaborated data sharing protocol, it would be possible to have a larger dataset resulting in increased processing capabilities especially with technologies that are powered by artificial intelligence (AI) tools. Through this way, as noted by Jiang et al. [35] and Allam [36], it would be possible to facilitate early detection, achieve better diagnosis and provide better urban management decisions for increased efficiency for virus containment. 

An example of how beneficial collaboration and sharing of data can be occurred during the 2014 Ebola outbreak in West Africa where scientists, health workers and clinicians, amongst other stakeholders from around the world, openly worked together and were able to contain the spread of this pandemic [37]. On this front, Boué et al. [38] highlight that levels of trust and transparency need to be reviewed and enhanced to facilitate unfettered data generation and sharing. Such could lead to an even earlier detection scenario of future virus outbreaks, and in the better curative management of the same, without minimal compromise on urban functions and on an urban economy.

Furthermore, in cases of emergencies like the current outbreak of COVID-19 and any other, the need for observance of regulatory practices and international healthcare guidelines are paramount. This would ensure that both healthcare professionals and the general populace are informed, protected and remain within the prescribed rules and regulations. As noted by the WHO [39], the healthcare guidelines and regulatory practices are advanced to also ensure that the health risk in question is reduced together with its consequences. In the current era of technological advancement, such regulations and guidelines are paramount as they have potential to lead to positive or negative outcomes. The position of this paper is to advance that it now possible to integrate technologies like the use of smart devices through IoT networks and wearable devices, data from mobile apps and others to help users to share information with accredited and certified health professionals, and in this case, improve the outcomes for better cross disciplinary and more resilient protocols and policies.

## 6. Conclusions

As the world increases in its ready adoption of the smart city concept, and its related technological tools, these tools need to be tailored to ensure that liveability dimensions are adequately catered for, including the thematic of urban health. On this front, it is argued that the lack of standardization between smart city technology suppliers can lead and is leading to non-communication between cities and data platforms. Such can, and is, resulting in a non-productive system in the case of virus outbreaks because early detection and management of the same can become increasingly dependent upon the technological backbone of smart cities. This paper thus highlights the urgent need to work towards the standardization of protocols for enhanced smart city communication and the need to democratize the smart city technology sphere to encourage equity and transparency amongst stakeholders, thereby providing more possible cooperation in the case of disasters.
